# Oxytocin and Vasopressin, and the GABA Developmental Shift During Labor and Birth: Friends or Foes?

**DOI:** 10.3389/fncel.2018.00254

**Published:** 2018-08-21

**Authors:** Yehezkel Ben-Ari

**Affiliations:** Neurochlore and Ben-Ari Institute of Neuroarcheology (IBEN), Marseille, France

**Keywords:** [Cl^−^]_i_, birth, labor stress, maternal behavior, social behavior, GABA developmental shift, neuroarcheology, excitatory inhibitory actions of GABA

## Abstract

Oxytocin (OT) and vasopressin (AVP) are usually associated with sociability and reduced stress for the former and antidiuretic agent associated with severe stress and pathological conditions for the latter. Both OT and AVP play major roles during labor and birth. Recent contradictory studies suggest that they might exert different roles on the GABA excitatory/inhibitory developmental shift. We reported (Tyzio et al., 2006) that at birth, OT exerts a neuro-protective action mediated by an abrupt reduction of intracellular chloride levels ([Cl^−^]_i_) that are high *in utero*, reinforcing GABAergic inhibition and modulating the generation of the first synchronized patterns of cortical networks. This reduction of [Cl^−^]_i_ levels is abolished in rodent models of Fragile X Syndrome and Autism Spectrum Disorders, and its restoration attenuates the severity of the pathological sequels, stressing the importance of the shift at birth (Tyzio et al., 2014). In contrast, Kaila and co-workers (Spoljaric et al., 2017) reported excitatory GABA actions before and after birth that are modulated by AVP but not by OT, challenging both the developmental shift and the roles of OT. Here, I analyze the differences between these studies and suggest that the ratio AVP/OT like that of excitatory/inhibitory GABA depend on stress and pathological conditions.

## Introduction

Labor and birth are amongst the most complex biological mechanisms in mammals. A series of vital adaptations must take place rapidly including the transition from a liquid medium to an aerial one (Bland et al., 1982; Hooper et al., 2016), a shift from continuous to intermittent nutrient supply, a drop in body temperature (Jäykkä and Laakso, 1967) and the release of catecholamine, glucocorticoids and Oxytocin (OT; Lagercrantz and Slotkin, 1986; Hillman et al., 2009, 2012). Noradrenaline and adrenaline levels rise within minutes of term birth and cord clamping to levels that are seldom observed even after severe stress (Lagercrantz and Bistoletti, 1977; Faxelius et al., 1983). These are important in the transition of fetal to neonatal circulation, and reducing pulmonary vascular resistance and increasing pulmonary blood flow (Aslan et al., 2008; Olver and Walters, 2008). Delivery is also stressful and potentially painful for the newborn (Lagercrantz and Bistoletti, 1977). Indeed, the “stress of being born” is important in triggering the breathing reaction that must take place at birth via a series of mechanisms that are beginning to be deciphered, including the squeezing of the fetus through the birth canal and the cold temperature outside the womb. Interestingly, high catecholamine levels due to fetal adrenal release (Newnham et al., 1984; Padbury et al., 1987) are lower in the un-labored fetus born by C-section but higher during vaginal delivery and even more so when associated with anoxic episodes (Greenough et al., 1987). Cortisol levels are increased dramatically and abruptly, from 5 μg/ml to 10 μg/ml at around 30 weeks of gestation, to about 45 μg/ml prior to labor at term, and to 200 μg/ml a few hours after term birth (Faxelius et al., 1983).

Clearly, birth is not just a continuation of fetal development but a unique process that calls for specific biological mechanisms aimed at preparing the brain to accommodate with the new environment. Birth is also a critical period with a large number of neurological and psychiatric disorders associated to birth-related complications including anoxic insults and preterm delivery (Ben-Ari, 2015). It is important to investigate whether and how is the brain prepared to labor and birth in naïve and pathological conditions. A Longitudinal study in humans suggest that growth is slowed down during the last 5–10 weeks of gestation suggesting that the brain is indeed preparing for delivery (Fujimura and Seryu, 1977). However, this study is not relevant to specific delivery related mechanisms as alterations shortly before and after vaginal delivery cannot readily be performed for ethical reasons in humans. Therefore, animal studies comparing physiological, electrical and morphological neuronal properties before and after delivery are indispensable in order to determine whether and how is the brain prepared for vaginal delivery. Astonishingly, very few such studies have been performed and they have been centered on the GABA depolarizing/hyperpolarizing and excitatory/inhibitory developmental shift (Tyzio et al., 2006; Spoljaric et al., 2017). These studies reported different roles of OT and vasopressin (AVP): two closely related neurohormones that have been preserved throughout evolution and play major and complementary roles in labor and birth. Here, I analyze the differences between these studies and propose a conceptual frame of operation of their actions in health and disease. However, I first briefly summarize their similarities and differences notably with regard to GABAergic inhibition in adults.

### OT, AVP and GABA: Interactions in Adults

Human and animal studies have shown that OT exerts major roles in social interactions (Insel, 2010). In contrast, the primary function of AVP is to maintain body fluid balance by keeping body and brain osmolality within narrow limits being released by thirst, endocrine and behavioral stress acting in life threatening conditions. Thus, AVP is involved in thermoregulation, cooling operating via activation of a non-selective cationic channel in AVP hypothalamic releasing neurons (Sharif-Naeini et al., 2008) and in response to hyperosmotic stress with high-salt diets induce or exacerbate hypertension (Kim et al., 2011; Choe et al., 2015; Prager-Khoutorsky et al., 2017). AVP receptor antagonists reduce stress and are candidates to treat affective disorders (Griebel et al., 2002). In prairie voles, OT but not AVP signals mediate social support reducing the effects of stress (Donovan et al., 2018). In general, AVP amplifies stress and the reactivity to stressors whereas OT reduces the amplitude of the stress response and improves emotion processing (Neumann and Landgraf, 2012; Iovino et al., 2018).

Intrinsic differences between interactions of OT or AVP with GABA signals are also well documented. Thus, hypothalamic AVP releasing neurons, have highly depolarized GABA reversal potential (E_GABA_; −33 mV) and depolarization after activation of chloride permeable GABA receptor-channels leads to neuronal excitation. In contrast, hypothalamic OT releasing neurons express KCC2, E_GABA_ is hyperpolarizing (−75 mV) and the hyperpolarization leads to neuronal inhibition (Haam et al., 2012; also see; Belenky et al., 2008). Opposite actions of AVP and OT are also documented in the amygdala where AVP excites and OT inhibits central amygdala neurons (Raggenbass, 2008) and in the hypothalamus where GABA exerts inhibitory and excitatory actions on OT and AVPO neurons respectively (Haam et al., 2012). Interestingly, high salt intake causes an increase of chloride levels ([Cl^−^]_i_) levels in AVP releasing neurons (involving a decrease of KCC2 and an activation of NKCC1), excitatory actions of GABA and AVP dependent hypertension (Kim et al., 2011; Prager-Khoutorsky et al., 2017). Therefore, AVP and OT are preferentially associated with excitatory and inhibitory actions of GABA, respectively.

### OT and AVP in Labor and Birth

#### Oxytocin: An Anti-stress Neuro-protective Agent That Reinforces Sociability and Mother-Baby Interactions

OT, whose levels rise sharply during delivery, exerts a multitude of functions including in analgesia, lactation and communication (Insel and Young, 2001), and anti-stress (Feldman et al., 2014, 2016; Murgatroyd et al., 2015; Sue Carter, 2018). OT signals also control fear expression in mothers lactating mice via projections of the hypothalamus to the lateral septum (Menon et al., 2018). OT increases maternal bonding in many animal species and humans (Feldman et al., 2014; Muscatelli et al., 2018). Infant macaques are positively influenced by OT that might underlie early social motivation (Simpson et al., 2014; Lefevre and Sirigu, 2016).

OT actions are also manifest in several pathological conditions. Thus, OT receptors are present on microglia and OT signals play an important anti-inflammatory action reducing the activation of microglia in pathological conditions (Clodi et al., 2008; Liu H. et al., 2016; Yuan et al., 2016). Blockade of OT signals during labor exerts many negative sequels (Simsek et al., 2012) but interestingly administration of OT to trigger/accelerate labor produces long term adverse sequels possibly reflecting the importance of local release (Boer and Kruisbrink, 1984; Wahl, 2004; Gregory et al., 2013). ASD-like behavior is observed in rodent knockout models of OT signals (Lee et al., 2008; Sala et al., 2013) and children with ASD have lower OT levels, OT signals correlating with parent–child communication (Modahl et al., 1998; Feldman et al., 2012). Therefore, alterations of OT signals during birth lead to long term sequels.

#### AVP Amplifies Stress in Labor and Birth

During labor and birth, the triggering stimuli and release conditions of AVP and OT are different (Haldar, 1970; Akerlund et al., 1999). Contrary to OT, AVP is thought to participate during the late labor and expulsion phases, rather that initiating labor *per se*. Plasma AVP levels are low during early phases but rise during active labor in sheep and are associated with an increased sensitivity after delivery (Stark et al., 1979). In addition, AVP release is hypothesized to increase in response to a decreased arterial pH and oxygen tension during the late phases of labor (Stark et al., 1979), and it’s increase occurs in response to hypoxia and hemorrhage (Clark and Silva, 1967; Haldar, 1970). Changes of water metabolism are opposite after AVP and OT administration (Boer and Kruisbrink, 1984).

Many stress agents including catecholamine and cortisol are released along with the activation of the hypothalamo-pituitary-adrenal axis (HPA) during labor and birth, and their levels correlate with stress severity, being for instance higher after vaginal than C-Section delivery, and more so following anoxic episodes (Greenough et al., 1987). The correlation between AVP or the biochemically stable C-terminal fragment of AVP copeptin is striking. Indeed, the release of AVP and copeptin is lower after C-section delivery and dramatically increased by birth asphyxia, therefore being associated with perinatal stress and acidosis (Schlapbach et al., 2011; Evers and Wellmann, 2016). Kaila and co-workers have recently shown that AVP (and copeptin) are excellent biomarkers of severe anoxic encephalopathy correlating with high lactate levels (Kelen et al., 2017). The authors suggest that “*copeptin and enolase measured in the early postnatal period are potential prognostic biomarkers of long-term neurodevelopmental outcome in term neonates diagnosed with HIE and treated with therapeutic hypothermia*”. AVP amplifies the reactivity to stressors and the stress response notably on amygdala neurons, with opposite actions on different amygdala nuclei (Stoop, 2012; Donovan et al., 2018; Iovino et al., 2018; Stacey et al., 2018). Therefore, AVP and OT are released by different stimuli, and exert different roles on labor and birth in naïve conditions and in pathological ones.

### The GABA Developmental Sequence: A General Rule That Has Been Preserved Throughout Evolution

The developmental sequence of GABA shifting from depolarizing to hyperpolarizing has been described in all animal species and brain structures investigated, and appears to have been preserved during evolution (Ben-Ari, 2002; Yamada et al., 2004; Ben-Ari et al., 2007; Chen and Kriegstein, 2015). In many, but not all conditions, this is associated with a parallel shift of the actions of GABA from excitatory (generation of sodium spikes) to inhibitory. However, the depolarization produced by GABA leads to an increase of intracellular calcium mediated by either the activation of voltage gated calcium currents and/or the removal of the voltage dependent Mg^++^ blockade of NMDA receptor channel mediated currents (Leinekugel et al., 1997). The unmasking of NMDA currents exerts a major role in immature systems and ought to be considered as a nodal feature of the GABA developmental shift. The Calcium rise generated by both mechanisms underlie the well-known trophic actions of GABA that exerts major roles on neuronal growth, synapse formation and cell assembly construction (Meier et al., 1983; Wolff et al., 1993; Represa and Ben-Ari, 2005; Kilb et al., 2013). Therefore, the activation of GABA signals exerts quite different effects in immature and adult neurons, although depolarizing and excitatory actions of GABA are also observed in adult neurons (Marty and Llano, 2005) and in a variety of pathological conditions (Ben-Ari et al., 2007; Kaila et al., 2014; Kahle et al., 2015). It bears stressing that the actions of GABA are also inhibitory due to the so-called shunting actions that co-exist with depolarizing or hyperpolarizing effects of the resting membrane potential (Khalilov et al., 1999). Therefore, GABA actions are highly suited to depolarize and unmask the conditionally silent NMDA receptors that are expressed before the AMPA type thereby facilitating developmental plasticity. In addition, because of the intrinsic shunting effect, the excitatory actions of GABA are not associated with the toxicity and the potentially dangerous actions of glutamatergic signals. This developmental shift in the action of GABA is mediated by an imbalance between the expression of the chloride importer NKCC1 and the chloride exporter KCC2, leading to higher intracellular chloride concentrations ([Cl^−^]_i_) in immature neurons (Rivera et al., 1999). The exact occurrence of the shift is brain structure and animal species dependent, further stressing the large heterogeneity of neurons during development.

### Oxytocin: A Neuroprotective Agent Reducing [Cl^−^]_i_ Levels at Birth

Relying on single NMDA and GABA channels to determine respectively the resting membrane potential (V_rest_) and GABA driving force (DF_GABA_), we reported an abrupt and important fall of [Cl^−^]_i_ levels shortly before and during birth (Tyzio et al., 2006). This drop starts and peaks before birth, ending shortly after birth. It appears to be mediated by OT signals (even though AVP was not tested), since a relatively selective OT receptor antagonist abolishes it. The effects of the OT antagonist was conspicuous at birth but not 2 days later, and the effects were abolished by intra-cardiac perfusion of the pup with saline solution suggesting the presence of endogenous neurohormone during a restricted period (Tyzio et al., 2006). Interestingly, OT signals modulate the generation of non-synapse driven voltage gated plateau potentials that are present prior to the synapse driven giant depolarizing potentials (GDPs), and blocked by maternal administration of the same OT receptor antagonist (Crépel et al., 2007). Whether the source of OT is the mother or the fetus has not been fully determined (Brown and Grattan, 2007).

We then showed that OT acting on a reduction of [Cl^−^]_i_ levels exerts a neuro-protective action on neurons reducing the effects of brief anoxic episodes, an observation that is in keeping with the well-known aggravation of the deleterious insults when ongoing activity is elevated (Dzhala et al., 2001; Nardou et al., 2011). Other studies also suggest neuro-protective actions of OT (Zinni et al., 2018). Interestingly, we reported that OT, like the NKCC1 antagonist bumetanide, reduced low [Cl^−^]_i_ levels in pain pathways thereby attenuating pain during birth (Mazzuca et al., 2011; also see; Gagnon et al., 2013; Chabwine et al., 2015; Eliava et al., 2016). This suggests an analgesic action of the hormone mediated also by [Cl^−^]_i_ levels regulation. This is in agreement with the lower pain experienced by babies born by vaginal delivery compared to babies delivered by C-section (Bergqvist et al., 2009). Direct interactions between OT signaling and [Cl^−^]_i_ levels via the chloride exporter KCC2 have been reported (Leonzino et al., 2016). Interestingly, the expression of GABA receptor alpha 1 subunits is elevated during pregnancy and falls at the time of parturition when OT neurons become active (Brussaard et al., 1997). Collectively, these observations suggest that in addition to its role in triggering labor, OT exerts a neuro-protective and analgesic role both involving reduction of [Cl^−^]_i_ levels (also see Camille Melón and Maguire, 2016).

The observed low [Cl^−^]_i_ levels and inhibitory GABA actions at birth were not anticipated because of the massively released stress molecules at this time (Lagercrantz and Bistoletti, 1977; Lagercrantz and Slotkin, 1986). Indeed, in rodents, exposure to a variety of stress protocols produces high catecholamine levels, down regulation of KCC2 and a depolarizing shift of GABA and neuronal hyperactivity (Wake et al., 2007; Hewitt et al., 2009; Sarkar et al., 2011; Inoue et al., 2013; MacKenzie and Maguire, 2013; Inoue and Bains, 2014; Maguire, 2014). This contradiction raises the possibility that during evolution, stress molecules were required to enable breathing and facilitate fast adaptations, but compensatory mechanisms were implemented to alleviate the deleterious effects of stress molecules on brain activity (Ben-Ari, 2015). At any rate, stress will strongly impact [Cl^−^]_i_ levels and GABA actions during labor and birth.

### The GABA Shift Is Abolished in Rodent Models of Autism, Fragile X Syndrome and Other Disorders

The regulation of [Cl^−^]_i_ levels is highly dynamic, being strongly dependent on the levels of neuronal activity. Thus in a wide range of brain disorders including epilepsies, brain trauma, cerebrovascular infarcts, spinal cord injury, autism and other developmental disorders, GABA exerts strong depolarizing and excitatory actions mediated by high [Cl^−^]_i_ levels and a down regulation of KCC2 and/or up regulation of NKCC1 (Kahle et al., 2015, 2016; Ben-Ari, 2017). We found that the depolarizing to hyperpolarizing shift at birth was abolished in the rodent models of autism (intrauterine Valproate administration) and Fragile X Syndrome (Tyzio et al., 2014). The persistence of these effects is suggested by the observation that excitatory actions are still present in hippocampal neurons 2 weeks later, suggesting that the polarity of GABA actions at this stage exert, by an as yet undetermined mechanism, long-term actions (He et al., 2014; Tyzio et al., 2014). Parallel observations suggest that this could be mediated by interactions between intracellular activation of OT signals and the chloride exporter KCC2 (Leonzino et al., 2016). Therefore, the GABA shift at birth is sensitive to pathogenic conditions. Interestingly, maternal restoration of low [Cl^−^]_i_ levels during labor and birth led to long-term attenuation of the electrical and behavioral features of ASD further illustrating the importance of GABA polarity at birth (Eftekhari et al., 2014; Tyzio et al., 2014).

In keeping with clinical observations (Atladóttir et al., 2009), Patterson and colleagues discovered that maternal immune activation (MIA) induce behavioral features of ASD in offspring (Patterson, 2011; Hsiao et al., 2013). Furthermore, Choi and colleagues have shown that MIA triggers a cascade of events including interleukin activation and microbiote modifications leading to abnormal cortical construction that is the final cause of the disease (Estes and McAllister, 2015; Choi et al., 2016; Kim et al., 2017). This “Neuro-archaeology” sequence (Ben-Ari, 2008) vividly illustrates the importance of intrauterine events in deviating developmental sequences that are the direct cause of the deleterious sequels. Interestingly, GABA exerts depolarizing and excitatory actions after MIA (Corradini et al., 2018). Depolarizing actions of GABA have also been observed in the 22q11.2 deletion syndrome where the excitatory to inhibitory GABA shift is abolished and restored by bumetanide in cortical neuronal cultures (Amin et al., 2017). The postnatal GABA shift is also reduced in mice with Down syndrome (Deidda et al., 2015) and many other pathological situations suggesting that bumetanide and similar agents might be the “aspirin” of brain disorders (Ben-Ari, 2017).

It is important to stress that [Cl^−^]_i_ levels measures even within the same laboratory are variable. Thus, we reported values extending from strongly hyperpolarizing to equipotential with V_rest_ and even slightly depolarizing (Tyzio et al., 2003, 2008; Chiesa et al., 2018). This most likely reflects the importance of external conditions, animal house conditions, stress, intrinsic permeability to HCO_3_. However, in the same experimental condition, [Cl^−^]_i_ levels are higher after insults or in pathological conditions, validating the hypothesis that [Cl^−^]_i_ is a marker of stress intensity. Therefore, in spite of its heterogeneity, [Cl^−^]_i_ levels provide a good indication of the pathogenic condition, with high levels at birth constituting a common signature of developmental disorders.

### AVP-Sensitive Excitatory Actions of GABA at Birth

Our understanding of the effects of AVP on immature neuronal activity is far more restricted than on those of OTs. Using rodents and guinea-pigs, Kaila and co-workers (Spoljaric et al., 2017) showed that the GABA analog isoguvacine excites hippocampal CA3 pyramidal neurons before and shortly after birth, generating sodium action potentials and activating voltage gated calcium currents thereby denying the occurrence of a shift. These excitatory actions were blocked by bumetanide suggesting high [Cl^−^]_i_ levels. Interestingly, AVP augmented the frequency of IPSCs in pyramidal neurons and reduced GDPs frequency suggesting an excitatory action of pyramidal neurons but an overall inhibitory effect on network activity. It is suggested that GABA excites neurons at birth- pyramidal neurons and interneurons- leading to a de-synchronization and an inhibition of network activity that impinge on principal cells consequently to that de-synchronization. These actions are mediated by AVP since they are blocked by a selective AV1 receptor but not by an OT antagonist, suggesting that AVP, and not OT, is the key player in this sequence. Therefore, the authors hypothesize that AVP exerts a neuro-protective action mediated by the desynchronization produced by excitation of pyramidal neurons and interneurons but there is no developmental shift of GABA actions, GABA exciting neurons before and after birth. Therefore, the contradictions between Tyzio et al. (2006) and Spoljaric et al. (2017) concern the roles of OT and AVP, the occurrence of the GABA inhibitory shift and the overall effects of GABAergic signaling on the network during labor and birth.

### Differences Between These Studies

Recording neurons *in utero* and shortly after birth is no simple task underlying why these studies are to the best if my knowledge the only ones performed *in vitro* (and *in vivo* as a matter of fact). There are several many possible causes for the discrepancies between Tyzio et al. (2006) and Spoljaric et al. (2017) considering the fragility of the tissues and the vulnerability of the fetuses and newborns. Below I am listing a series of technical differences between the studies.

#### Anesthetic Agents Impact GABAergic Signals

In our studies, acute hippocampal slices were prepared from fetuses or neonatal pups following a decapitation without anesthesia. Kaila and colleagues (Spoljaric et al., 2017) used anesthetic agents (notably volatile anesthetic agents) in most of their experiments. The use of anesthetic agents might hamper the conclusions notably on fetuses as the peak GABA hyperpolarizing shift occurs shortly before birth (Tyzio et al., 2006). Indeed, they exert a plethora of effects notably silencing cortical neuron, increasing [Cl^−^]_i_ levels and corticosterone in a bumetanide sensitive manner, and shifting the polarity of GABA actions notably during the early post-natal period (Zaninetti and Raggenbass, 2000; Owen et al., 2013; Lim et al., 2014; Xu et al., 2015; Harden and Frazier, 2016; Liu G. et al., 2016; Martynyuk et al., 2017). They increase caspase3 and aldosterone serum levels, effects that are attenuated by OT (Cao et al., 2012) and impact hippocampal networks (Raggenbass, 2008; MacIver, 2014; Brohan and Goudra, 2017; Kuo and Leung, 2017) with short- and long-term alterations (Wrobel et al., 2010; Khanna et al., 2013). Sevoflurane is toxic and inflammatory in babies and toddlers (Juif and Poisbeau, 2013; Sitdikova et al., 2014; Whitaker et al., 2017; also see Zaninetti and Raggenbass, 2000; Raggenbass, 2008; Wrobel et al., 2010; Khanna et al., 2013; Lim et al., 2014; MacIver, 2014; Liu H. et al., 2016; Alvarado et al., 2017; Brohan and Goudra, 2017; Cornelissen et al., 2017; Kuo and Leung, 2017; Perez-Zoghbi et al., 2017; Stolwijk et al., 2017). Interestingly, sevoflurane down regulates release of OT and AVP, impairing social interactions (Zhou et al., 2015). These effects are long lasting with persistent sequels (Andropoulos, 2018; Raper et al., 2018). These differences preclude comparing the actions of GABA between these studies.

#### Recording Techniques and Excitatory Actions of GABA

To determine genuinely V rest and the driving force of GABA, we relied on single NMDA or GABA channel recordings, respectively that are a necessary technique needed to avoid alterations of resting membrane potential and to provide reliable measures of [Cl^−^]_i_ levels (Tyzio et al., 2003). Spoljaric did not determine directly V rest and the technique used to measure [Cl^−^]_i_ levels are indirect, it is therefore difficult to compare the genuine values of V rest and DF GABA.

#### Excitatory or Inhibitory Actions of GABA

To determine the effects of GABA, Tyzio et al. (2006) used low concentrations of the GABA_A_ receptor agonist isoguvacine (10 μM) while Spoljaric et al. (2017) used 10-fold higher concentrations (100 μM) raising the possibility of effects on intrinsic properties including sodium channel inactivation, suppression of firing and an after-inhibition rebound that can be considered as an excitatory action of GABA (Haam et al., 2012).

#### Ongoing Activity

In our hands, bona fide GDPs (>0.5 s, 0.1–0.5 Hz) are rarely observed at birth (and none before birth) and there is a low frequency ongoing spike activity that is abolished by GABA/Glutamate receptor antagonists (Ben-Ari et al., 1989). This differs significantly from the observations of Kaila and colleagues with higher ongoing activity and GDPs (Spoljaric et al., 2017) suggesting differences in rodent strains or conditions between the French and Finnish laboratories. We recorded frequently calcium plateau potentials (SPAs; Crépel et al., 2007) which were apparently not recorded by Spoljaric et al. (2017).

#### AVP Presence in the Hippocampus Identified by Immunocytochemistry

Kaila and colleagues suggest that AVP is released in the hippocampus relying on immuno-cytochemical observations. However, the Neurophysin antibody (NP AVP 41) does not dissociate AVP from prohormone (Ben-Barak et al., 1985; Whitnall et al., 1985).

#### OT and AVP

Considering the complex interactions between OT and AVP signals and their inter-changeability, it is difficult to ensure that the peptides are acting only on their receptors and selectively blocking OT or AVP receptors does not necessarily imply that this is the cognate ligand. Thus, OT still exert its actions when OTRs are blocked through V_1a_ AVP receptors and SR49059 also blocks OT receptors (). Clearly, further studies are needed to dissociate the actions of OT and AVP signals that have not been compellingly differentiated by both groups.

### OT and AVP Actions on GABA at Birth

Beyond these different technical issues, the principal conceptual issue debated is whether labor and birth impact ongoing electrical activity. The extremely large pathological conditions associated with high [Cl^−^]_i_ levels suggest that this is a signature of stress and neuronal vulnerability (Ben-Ari, 2017). The magnitude of these changes is animal species and brain structure dependent. I suggest that during late labor and birth, OT provide a counter signal to stressors molecules including cortisol, catecholamine and AVP. This is mediated by a reduction of [Cl^−^]_i_ levels that these agents tend to augment producing a hyperpolarizing GABA shift and a reduction of ongoing activity. I suggest as a working hypothesis that the AVP/OT ratio like the GABA depolarizing to hyperpolarizing shift during labor and birth depend on the severity of stress, pain, asphyxia, anesthesia and catecholamine-cortisol release. With moderate stress, in addition to inducing labor, OT signaling intervenes acting to develop maternal-infant bonds and reduce ongoing electrical activity around birth. During more severe intrauterine stress, AVP is released (Stark et al., 1979) exerting its natriuretic action and preserving water balance. In this perspective, the excitatory actions of GABA are viewed as a collateral effect in spite of its possible deleterious actions on brain activity but also a signature of this potentially pathogenic condition. This situation is not unprecedented. GABA exerts different effects notably on transmitter release in chromaffin cells. GABA exerts opposite effects in moderate and severe stress on catecholamine or acetylcholine release (Kataoka et al., 1984; Alejandre-García et al., 2018), a situation that is reminiscent of immature neurons (Harada et al., 2016). Similarly, GABA exerts diurnal day/night shifts of actions in the suprachiasmatic nucleus (Wagner et al., 1997; Irwin and Allen, 2009). Clearly, the dynamic feature of GABA actions and the shift of its polarity provide a unique mechanism to regulate activity dependent mechanisms during conditions of stress and high degree of vulnerability.

## Conclusion

The dual facets of the actions of GABA and its exquisite dependence on the overall activity and on [Cl^−^]_i_, underlie the actions of OT and AVP on ongoing activity, and taking into account external factors including the magnitude of stress. These observations have also an important impact on our understanding of the possible pathogenic events taking place during labor and birth. They necessitate more investigation on the links between stress molecules and OT/AVP actions during birth and a systematic evaluation of their effects on GABA actions. Alterations during the perinatal period produced by pathological conditions could be therefore hypothesized to be associated with enhanced actions of AVP over OT signals and excitatory GABA actions over inhibitory ones.

## Author Contributions

The author confirms being the sole contributor of this work and approved it for publication.

## Conflict of Interest Statement

YB-A is a share holder and CEO of Neurochlore.
